# Life story templates in dementia care: Ambiguous direction and purpose

**DOI:** 10.1177/14713012231224545

**Published:** 2024-01-04

**Authors:** Glenn Möllergren, Tove Harnett

**Affiliations:** School of Social Work, 166452 Lund University, Sweden

**Keywords:** dementia, life stories, life story work, life story templates, biography

## Abstract

**Background:**

The use of life stories in dementia care has been described as a way of seeing every person as an individual, looking beyond their dementia. Life stories have become synonymous with high-quality care, while in Sweden their mere existence in dementia care settings is taken to indicate quality in national comparisons. Such life stories are often standardised, generated by a family member answering predetermined questions in a template.

**Aim and methods:**

Using a constructionist approach, this study will (1) chart what versions of a person’s life story the templates produce, and (2) establish the intended purpose of such life stories, as communicated by the templates. This study departs from the assumption that life story templates communicate something about the conceptions of people living with dementia. The thematic analysis used data comprising 30 blank templates, totalling about 1,700 questions.

**Findings:**

The life story templates were found to generate two very different versions of the individual: (1) a person before symptoms of dementia or (2) a patient *with* dementia. We also found contradictions about what information should be included, whose life story it was, and the intended use.

**Discussion:**

Despite strong pressure on dementia care providers to collect life stories from residents, the life story templates they use are without clear direction, ideology, or purpose. The lack of direction is key given that life stories can be considered actants that shape assumptions about people with dementia and construct realities in dementia care settings. We highlight the need to develop ethical guidelines for life story template design, matched with guidelines for their intended use.

## Introduction

People who move into a dementia care home may experience a range of threats to their identity and sense of self. The progression of a cognitive decline may undermine their sense of identity and other people may perceive them as entirely different to how they were before (Caddell & Clare, 2011; Enright et al., 2020). From their family members’ point of view, the communal nature of life in a dementia care home may be thought to reinforce this process, and there is a documented fear that dementia care settings deprive residents their unique identities (Harnett & Jönson, 2017).

To counteract this depersonalisation, dementia care homes in many parts of the world have adopted person-centred care, including the incorporation of life stories (Ejaz et al., 2022; Kindell et al., 2014; Socialstyrelsen, 2017; Stranz & Sörensdotter, 2016). Life stories are often considered to maintain threatened identities (McKinney, 2017) so that others ‘see’ and understand the person behind the dementia (Clarke et al., 2003; Cooney & O’Shea, 2019; Edvardsson et al., 2010; Kitwood, 1997; McKeown et al., 2010; Sweeney et al., 2021). When the British Department of Health rolled out life story training for dementia care staff, the Secretary of State for Health and Social Care described it as a way to ‘see every person as an individual, looking beyond their dementia’ (Dementia Partnerships, 2011). Life story work has been described as ‘synonymous with best quality care’ (Baillie & Thomas, 2020, p. 575) and it has been promoted by organisations such as Dementia UK (2023) and Diakoneo (2019) in Germany. Life story work is included in Swedish eldercare policy and is one of the quality indicators for rating dementia care homes (Socialstyrelsen, 2018). Socialstyrelsen, the Swedish National Board of Health and Welfare, gives the reasons:

The life story can have a description of important life events and of what a person considers important. The information can give a sense of who the person is and has been. This can provide guidance and give professionals the possibility to take the perspective of the person with dementia and how she or he might understand the world. (Socialstyrelsen, 2019, p. 34, p. 34)

Preserving one’s sense of identity, Kitwood (1997) states, is key for people living with dementia, and because ‘it involves a sense of continuity with the past’ (p. 21), care staff should be provided with biographical information about the people they care for. Kitwood argues that not only could life stories be integral to person-centred care by maintaining individuals’ identities, but also that they could create an environment where personal growth is possible.

Academic interest has followed on the increase in life story work in eldercare. A review of the literature shows the majority of studies focus on the importance of life story work as a phenomenon (Grøndahl et al., 2017), while only a few studies consider how life stories are used by staff (Clarke et al., 2003; Moos & Björn, 2006), their impact (Karlsson et al., 2014), or their usefulness to family members (McKeown et al., 2010). Similarly, an absence of life milestones such as marriage has been described as potentially crucial for life story work and identity construction (Blix et al., 2015).

The aim of this study is twofold: (1) to chart what versions of a person are produced by life story templates, and (2) to establish the intended purpose of such life stories, as communicated by the templates. Specific attention has been paid to the assumptions which life story templates communicate about people with dementia.

## Life story work in dementia care homes

Life stories are collected in eldercare contexts worldwide, including dementia care (Grøndahl, et al. (2017). Some researchers consider life stories are a way of evaluating actions and creating meaning (Atkinson, 2007; Hoey, 2005), while others believe they reveal the true person assumed to be ‘hiding’ inside the shell of dementia (Cooney & O’Shea, 2019, p. 2742). Four main benefits are commonly mentioned in research on life stories, reflecting the different understandings (Gridley et al., 2016; McKeown et al., 2010): reminiscence; a shift in focus from patient to person; managing trauma and anxiety; and tailoring daily care.

### Reminiscence

The first benefit is reminiscence. Kaiser and Eley (Grimwade, 2018, p. 21) relate life story work to the well-known activity of ‘reminiscence and putting one’s life in perspective’ by creating a life story book, memory box, digital story, etc., which is considered a largely therapeutic intervention which can be beneficial, no matter how the life story document is later used by care staff. This is supported by a large study by Subramaniam et al. (2014) which concludes that individual reminiscence work, using a life review or life story process, has potential psychosocial benefits for people with dementia. Others have found that putting the story together is enjoyable and fun (McKeown et al., 2010) and can be helpful for family members when someone dear to them lives with dementia (Andersson et al., 2019).

### From patient to person

The second benefit is a shift in focus ‘from patient to person’. Life story work can help change care staff and others’ attitudes towards a person with dementia (Grimwade, 2018, p. 25) because it encourages them to adopt a ‘compassionate culture’ and avoid the poor care – ‘degrading treatment’ – that results from ‘losing touch with some of our most fundamental values’ (p. 26). By carrying out life story work, people with dementia will grow in the eyes of others into rounded individuals whose values, needs, preferences, and desires are hard (er) to neglect. Ebert et al. (2020) find that ‘personhood-based knowledge’ increases social comfort for people living with dementia, because it forces care providers and others to overcome their ‘dementia fear’ (2020, p. 2550). McKeown et al. (2010, p. 153) term this as going ‘from patient to person’. This argument in favour of life story work centres on improving the social environment by using life story knowledge to achieve more respectful, tolerant behaviour towards people living with dementia.

### Trauma and anxiety

A third benefit is the better management of anxiety. Staff knowledge about the people living with dementia and their life stories has been described as a cornerstone in this effort. When staff have facts about a client’s past they may be inspired to chat about old times, which can have a calming effect on someone feeling ‘lost in the present’ (Edvardsson & Nordvall, 2008, p. 494) and can reinforce a sense of identity in a person affected by memory loss and anxious because of it (Clarke et al., 2003). Descriptions of a resident’s childhood can be an opportunity for staff to talk about the past and for the resident to gain a sense of control (Beechem et al., 1998). Biographical knowledge may also offer guidance and understanding at times of insecurity and suffering (Goyal et al., 2019) because such emotions can derive from trauma or loss experienced earlier in life (Gridley et al., 2020), which makes it crucial to obtain background knowledge about care recipients.

### Daily habits and routines

A fourth benefit is tailoring daily care to individual preferences and habits. Berendonk and Caine (2019) find that the further dementia progresses, the more life story work focuses on handling daily life in a pragmatic way – choice of food, daily routines, clothes etc. (p. 288). Thus this fourth benefit of life story work revolves around practical daily care (Baillie & Thomas, 2020; Brossard Saxell et al., 2021; Kitwood, 1997).

### Templates as actants

While people in the early stages of dementia may be involved in telling or writing down their life story, in the later stages people may find it too challenging and it falls to relatives or staff. This is most likely what happens in Swedish dementia care homes, since only people with extensive cognitive impairment are eligible for a place (Björk et al., 2016; SOU 2020:80; Socialstyrelsen, 2014; Starr & Szebehely, 2017) so when they move in the writing of their life story will require the involvement of relatives or staff (Andersson et al., 2019). To help families to write life stories, a rich flora of life story templates and booklets are being produced (Kindell et al., 2014) and these templates are the focus of this study.

Drawing on Holstein’s work (1992), we hold life story templates to be agential: they shape perceptions and the realities of people with dementia who live in dementia care homes (Atkinson & Coffey, 2011). Sweden has 290 local authorities, all with significant self-governance. Eldercare is a local authority responsibility and many local authorities and private care providers have developed their own life story templates. These consist of written, open-ended questions to be filled in by family members or by the older people themselves, and which shape the kind of life story that unfolds. As with other documents, life story templates impact the people who use them, suggesting interpretations and excluding others (Kelle et al., 2015). Documents, Holstein (1992) says, shape and manage reality and can be analysed by focusing on their use in human services, where they ‘produce’ the clients so that they fit with what the organisation can offer. Swedish life story templates are meant to give ‘professionals the possibility to take the perspective of the person with dementia’ (Socialstyrelsen, 2019, p. 34) and make assumptions about these people’s identities.

By studying life story templates, this article positions itself in a growing field of research about how documents shape professionals’ perceptions of clients and patients (Barfoed, 2019; Gillingham, 2016; Hjärpe, 2020). Guided by this constructionist approach, we understand life story templates as vehicles for instructions or scripts for how professionals in dementia care homes should perceive and treat the people they care for.

## Method and data

This study is part of a larger research project, Life Stories in Eldercare: Challenges and Possibilities (financed by Ribbingska Minnesfonden). This article is the first of two parts of the project; the second, yet to be reported, is a study of focus group interviews where care staff discuss their use of life stories in daily care work.

Data for the first phase was collected from May to July 2021. It consisted of life story templates from 28 local authorities, the dementia association Memorybond, along with Aidville and Wardday, two large for-profit care providers in Sweden. Of Sweden’s 290 local authorities, 30 were selected as representing large urban areas, suburban towns, and rural communities, using a balanced randomised choice of 10% of the total. The 30 were approached with an initial question about whether they use life story templates in their dementia care, which proved to be the case for all but one. Two did not reply, and one uses Memorybond’s template. The empirical data thus consists of 30 electronic documents. Data was thus based on publicly available documents and involved no human subjects or sensitive personal data. Research was conducted in accordance with the Helsinki Declaration and the Ethical Review Act (2003:460) stipulates that no ethical approval was needed. Care providers’ names have been fictionalised.

The life story templates studied here varied considerably in size and appearance, and most were apparently designed to be printed out, offering blank lines or boxes after each question, to be filled in by hand. Of the 30 templates, 22 featured an introduction, explaining the reasons for documenting the life story. All 30 templates asked for basic personal data, such as the name of the person and their family members. The fact that all the selected templates have been taken from one country is a limitation, given that in Sweden only people with extensive cognitive impairment are eligible for care in dementia care homes; however, in other countries too it is common for family members to fill out any life story forms.

The length of the templates in our sample ranged from 8 to 148 questions and from 3 to 30 pages. The data set thus totalled at least 285 pages and 1,700 questions. Many questions were formulated as sentences with several questions at once; in other instances, templates provided boxes to tick according to choice. To approximate the total number, such examples have been rounded down rather than up. Some life story templates had a colour front page, with images of flowers, meadows, hearts, hands, and lush trees, an old piano or a blue sky with white clouds; others were black and white. Some templates resembled that of Alzheimer’s WA (2017), because they had several pages of questions for or about the person with dementia, with blank lines for the answers.

All the templates were read independently by both authors of this article. Adopting a constructionist approach, we focused on the assumptions about people with dementia as mediated by the templates. A thematic analysis was conducted first to code the questions by theme, informed by Braun and Clarke (2006), who argue that a set of ‘themes ‘emerging’ or being ‘discovered’ is a passive account of the process of analysis’ (p. 7) and researchers should instead actively examine their data to define meaningful themes. In order to ensure the credibility of our findings, we were guided by the principles of analytic induction (Katz, 2001; Silverman, 2006), an exclusively qualitative strategy that seeks encounters with new varieties of data in order to force the revisions which make the analysis valid when applied to an increasingly diverse range of cases (Katz, 2001). Our analysis thus gradually deepened as we identified how the templates produced new versions of their intended purpose and the assumptions that life story templates communicated about people with dementia.

The resultant themes were then sorted according to whether they focused on the person *with* or the person *behind* the dementia diagnosis. To gauge whom the template was intended for – the person living with dementia, their family members, or professionals – we then read a second time for descriptive information such as the number of questions asked and whether the data collected was first person (‘*My* memories from school’, ‘TV programmes *I* like’), second person (‘*Your* memories from school’), or third person (‘*His* or *her* memories from school’). The second reading focused on how different questions presented the person with dementia in a different light. We also investigated how the author presented the stated reason for the template to users. A third reading followed to look for less common questions, such as traumatic life events, abuse, sex, and interpersonal relations.

## Findings

The analysis found the five themes discussed below. The first was the ambiguous function of the life stories. The second and third themes were the two versions of people that unfolded through the templates: as a patient *with* dementia or a person *before* symptoms of dementia. The fourth theme was the assumption that views and wishes are stable over the life course. The fifth and final theme was that templates were based on the assumption that people had led happy, unproblematic lives. The key findings will be described here using excerpts from the templates.

### Ambiguous intended purpose of life stories

Most templates had a rubric that encouraged the reader to complete the template and explained why a life story was important. Here we identified various promotional elements, such as ‘Your life is important and has a great impact on everyone around you!’ (Gardenbay local authority) or that a life story helps the staff to know ‘what brings a silver lining to your day’ (Millbrook local authority). The life story was promoted as something that will have an impact on ‘your life’ and ‘your day’. An intimate genre is constructed in parts of the text which describe how the life story not only provides information, but also helps ‘us’ understand and make ‘you’ feel more at home:

Your story is a tool that helps us get to know and understand you better by giving us a glimpse into your life. This way, we know how you want things to be in the future and can provide you with the best possible quality of life. (Riverwood local authority)

The personalisation of the reader (you/yours) and the care setting (we/us) simulate a personal relationship where both parties are helping each other. ‘You’ help the staff by completing the form; the staff helps ‘you’ by understanding the way you think.

The analysis revealed a great variation in assumptions about the intended author and mixed messages about whom the life story belonged to. Eleven templates were in the first person, seven in the second or third person, and thirteen templates used two or more. The title page communicated the same variety. The use of the first person for the document title suggested it was the person themself writing their life story:

Get to know me. My life story. (Forestville, Lindenhall, and Crystalport municipalities)

My life. My story. (Millbrook local authority)

I especially prefer these dishes. (Islehaven local authority)

Presuppositions are words or phrases that are a tacit assumption of the truth of a statement. When a life story template uses the phrase ‘Worth knowing about me’ (Ashkilltown local authority) it is presupposed that ‘me’ is the person with dementia and that they will have filled in their life story themself. Eleven of the templates used formulations such as:

This is how I want to be greeted. (Oliverstream local authority)

This makes me happy. (Oliverstream local authority)

At the same time, it is well known that family members fill in the vast majority of life story forms in Swedish dementia care settings. This highlights the ethical issues of whose story it is and how family members deal with the disclosure of material the person with dementia might not want to have recorded or shared with a wider audience (McKeown et al., 2015). Another formulation was the use of the second person, where the templates used the pronoun ‘you’ to address the intended author. This was also seen in the background information about the reasons for having a written life story: ‘A life story is a description of your life’ (Lydencove). As with the first-person writing, using the second person in headings and questions assumes that the life story will be written by the person with dementia, not a family member:

Your life story. (Guardhill local authority)

Life story. You know. (Cordhaven local authority)

Do you have any special traditions? (Hedgerow local authority)

The questions the templates asked added to the ambiguities in pronouns. Thirteen templates mixed formulations containing ‘I’ with those using ‘you’, ‘s/he’ or a passive form. Of the remaining eighteen, eleven used ‘I’. Another version was writing in the third person, with pronouns such as ‘she’ or ‘he’.

Childhood home and other known addresses where s/he has lived. (Carrickham local authority)

This kind of formulation assumed a family member or someone else with personal knowledge would fill out the person’s life story form. This ambiguity about relationships was also evident in phrases about the purpose of the life story. One template described it as:

The document about me. A description of me and what I want my life to be like when I can no longer speak for myself. (Islehaven local authority)

Other templates stressed the importance of people’s past by using the third person to explain the purpose of the life story: ‘Because the staff need to know the individual’s background’ (Aspenharbor). Some local authorities focused instead on the staff’s situation:

From the life story, we, as staff, can get to know the person from insight into his/her life. (…) What the person’s habits are. What food he or she prefers, and so on. Using this knowledge, we hope it will bring a silver lining to the individual’s life. (Carrickham local authority)

The templates made a variety of assumptions about what should be known about a person’s life and what not, and indeed about the intended author and owner of the life story. Some templates focused almost exclusively the present care situation, but others focused on the person’s past and childhood. Most templates had a rubric or introduction, but they differed considerably, with some describing the life story as the property of the person living with dementia, while others stated that the completed life story form belonged to the care provider.

### Version produced by the template: A patient with dementia

In six life story templates, some two-thirds of all the questions asked for information about the person’s current care needs, preferences, and habits, and by doing so constructed people predominantly as *patients* and as their situation ‘in the present’. One example was the template from the Islehaven local authority, which had 2 initial questions about ‘upbringing’ and ‘working life’, followed by 35 questions about current preferences in such things as nail care, music, TV shows, and aftershave. Information about food allergies, hygiene habits, sleep patterns, and TV and radio preferences can easily be transferred to a care context, and templates such as the one from Islehaven are essentially care scripts, asking questions such as:

This is how I like my hair to be styled. (Islehaven local authority)

I want my make-up like this. (Islehaven local authority)

I wear the following perfume. (Islehaven local authority)

I like to wear the following jewellery. (Islehaven local authority)

I don’t like this kind of music. (Islehaven local authority)

This makes me happy. (Islehaven local authority)

Activities that are interesting at the moment. (Carrickham local authority)

Important to note regarding personal hygiene, shower, and bath. (Wardday corporation)

Which activities I can/want to still engage in. (Crystalport local authority)

Favourite food? Something you don’t like? Do you have any allergies? How do you take your coffee or tea: milk and sugar or black? Wine, a schnapps with herring, or do you abstain from alcohol? Smoking or moist snuff? (Nesslake local authority)

These questions relate to the fourth type of benefit of life story work, intended to refine care scripts to personalise daily care.

### Version produced by the template: A person before symptoms of dementia

While all templates had at least a couple of questions about parents and siblings and the person’s childhood and school, four had a far stronger bias towards the ‘person before symptoms of dementia, with over 80% of questions about biographical data. Umbermouth local authority’s life story template, for example, exclusively asked about the past and was based on open-ended questions grouped by themes such as personal stories and memories, childhood and upbringing, adulthood, and place of origin and homes. While information about food allergies, hygiene habits, and TV preferences are easily transferred to a care context, the themes about past memories are more difficult to translate into daily care interventions.

Past research on life stories describes staff seeing them as tools that may generate emotionally meaningful situations for people living with dementia, but also as tools for collecting ‘true facts’ about residents’ pasts (Berendonk & Caine, 2019). Using the classification presented earlier, reminiscence-oriented templates may be linked to the first three categories: to provide a tool for reminiscence work for its own sake; to reduce the risk of maltreatment stemming from poor personal knowledge on the part of care staff; or to create a resource for handling anxiety using biographical data in the care situation. These are different in approach than a care script-orientated template.

The theme ‘Adulthood’ in Umbermouth local authority’s template asked about ‘stories and memories from work, occupation (for instance, worked from home, attitudes to work, enjoyed work, or wished had chosen a different education)’. The theme ‘Personal stories, memories, and context’ asked for greater detail about life before the dementia symptoms occurred:

Atmosphere in your childhood home. (Umbermouth local authority)

What were your mother and father like? (Umbermouth local authority)

Name some important life events. (Umbermouth local authority)

What was your profession? (Umbermouth local authority)

What hobbies and interests have you had? (Umbermouth local authority)

What in life has brought you happiness and joy? (Umbermouth local authority)

Even though none of the questions in Umbermouth local authority’s template related to the care situation, one of the last questions – ‘Thoughts on life, the end of life, and death’ – was indirectly linked to the care setting, as it was likely to be the place where the person lived until they died. What became obvious from the analysis was that the Umbermouth and Islehaven local authority templates mediated completely different assumptions about the same individual. If the person lived in a care home in Umbermouth, the life story would reproduce a person from the past, with a life course that ends when dementia sets in. If the person lived in Islehaven, however, the life story would be built on the person’s present-day preferences and may disconnect him or her from earlier roles and relations.

### Assumptions communicated about people with dementia: Static views

All but five templates asked questions about ‘personality’, understood as something stable across the life course. While 25 out of the 30 templates asked for data on ‘personality’, a majority limited themselves to only one such question. These questions were often accompanied by sub-questions about political, religious, or philosophical beliefs.

Questions about ‘personality’ typically came with possible answers such as ‘talkative’, ‘shy’, ‘sociable’, or ‘stubborn’. Half of the templates had follow-on questions about religious or ideological values and traditions, and some also asked about memberships, organisations, and political affiliations. Typical personality-oriented questions were:

Personality (talkative, shy, sociable, thrifty, etc.). (Aspenharbor local authority)

Personality throughout life (…) such as talkative, quiet, reflecting, worried. (Lindenhall local authority)

Distinctive personality traits, e.g., happy, brooding, social, shy, wanted to be alone, careful with money, generous, orderly, etc. (Aidville corporation)

Your typical personality traits (short-tempered, patient, cheerful, positive, depressed, cautious, accurate, etc.). (Memorybond association)

While it being valuable for care staff to understand individuals’ different personalities, these questions communicated values and views as something fixed and stable through life.

### Assumptions communicated about people with dementia: Having an unproblematic life

People living with dementia may relive traumatic or difficult life events as if they are taking place here and now. Knowing about such life experiences can help staff find a suitable approach. However, our study reveals a strong bias in the life story templates towards information about positive or neutral areas of the care recipients’ lives. In four cases, the explicit purpose of the life story, as stated in the template, was described as ‘bringing a silver lining’:

The life story serves as a resource for staff to ensure you are cared for according to your wishes and to bring a silver lining. (Millbrook local authority)

Even though eighteen templates had at least one question about ‘great grief’, only one asked specifically about the person’s experience of trauma. Questions about grief or suffering often included both ‘joy and grief’, leaving it up to the person to choose whether to write about joyful or sad life events. Six templates mentioned suffering and grief more than once, while twelve made no reference to sad, traumatic or problematic life experiences but asked at length about joyful memories and aspects assumed to provide people with happiness and pleasure.

The analysis also shed light on what was absent from the life story templates. Very few asked about mental health problems, substance use, abuse, violence, trauma, or sexuality and gender. Only one template had a question about the client’s sexuality. Questions about spouses and partners during the life course rarely provided space to name more than one spouse or partner and were invariably referred to in the singular.

The largely positive focus, omitting very personal information, sheds light on the ethical challenges of designing life story templates. While information about trauma and grief may be helpful for staff in providing person-centred care, it has ethical implications. Previous studies have shown that even people who are willing to verbally share their private, intimate memories may not want anything written down in a life story and being public knowledge (McKeown et al., 2015). As noted, ethical issues of this kind relate to the ambiguity about the purpose of life stories and whom a life story belongs to.

## Discussion

Swedish care providers’ life story templates vary widely in terms of what ‘version’ of the person they produce, who the life story belongs to, and its intended function. Top-down governance requires that care homes have life stories, but there is no consensus about what a life story is or what information it should cover (Bruner, 2004; Gridley et al., 2020; Hydén, 1997).

We find the life story templates produced two main versions of the people they were about: (1) a *person before* the dementia symptoms occurred or (2) a *patient with* dementia. The first can be linked to the first three benefits of life story work described in the literature (reminiscence, a shift in focus from patient to person, managing trauma and anxiety) while the second ideal aligns with fourth benefit (tailoring daily care).

Following Holstein (1992), it seems reasonable to say that templates which focus on the time before the onset of dementia will reproduce a person from the past, with a life course that ends when dementia sets in. As carriers of instructions, or scripts for staff, these templates provide guidance for reminiscence, rendering invisible the key transition in residents’ lives, that of getting dementia (Berendonk & Caine, 2019). Life stories that only focus on a person’s past, before cognitive decline, are valuable for seeing the person behind the dementia diagnosis, but may risk locking people in as fixed and unresponsive to change (Wellin & Jaffe, 2004).

Templates with a heavy focus on the present risk, by contrast, reproduce people predominantly as care recipients or patients and as people with dementia. A life story built on a resident’s present-day preferences may be useful for care staff on a daily basis, but risks disconnecting individuals from their biography and from earlier roles and relations. These templates provide little guidance for handling anxiety connected to trauma or negative life events in the past.

The life story templates were heavily skewed to unproblematic areas of life, and excluded questions about mental illness, substance use, abuse, and relational problems. The open-ended character of several questions did allow care recipients to write about problematic life experiences (when a template asked about ‘important life events’ or ‘atmosphere in your childhood home’, care recipients or family members had the chance to tell stories of abuse or trauma), yet, viewed as a vehicle of instruction, the templates risked (re)producing problem-free people with problem-free pasts. As we have seen, some care providers’ templates elicited responses free of life experience and biography, instead promoting lifestyle choices, personal taste, and cultural belonging. Others reproduced a historical person whose life was already over, and who had no ambitions in their current life. While in many ways contradictory, both of these ideal types are attuned to ‘delivering’ manageable clients to the care system. Specific tastes in food, music or clothing can in principle be handled in modern dementia care. It is possible to enjoy a glass of wine or take sugar in your coffee. Similarly, care recipients’ need to chat about old times, childhood memories, or their working lives may be met by care providers, insofar as they have obtained the knowledge required.

Life story templates can be useful prompts when people move into care homes, whether they write them themselves or with the help of a family member. Yet while ostensibly neutral, this article shows that life story templates can be actants that shape how people with dementia are reproduced, concealing experiences and problems that may be important in people’s lives. Dementia care homes are expected to collect life stories, but our study shows there is a lack of direction and purpose in what risks being no more than a paper exercise.

### Implications

In the light of our findings, we would propose reforming work with life stories in dementia care in Sweden in such a way as to develop a comprehensive method or guide by a leading stakeholder such as Socialstyrelsen (the Swedish National Board of Health and Welfare). This guide should be grounded in research about how people with dementia experience care that incorporates life stories. The design of future life story templates should be founded on a robust body of knowledge, thus ensuring they are not only person-centric but also adaptable to the unique life experiences of each individual living with dementia, including problematic life events.

Such a guide would serve as a practical tool for care providers and emphasise the importance of preserving the personhood of individuals with dementia by maintaining a sense of continuity with the past and fostering an environment conducive to personal growth. Additionally, the guide would address the challenges of creating a life story: the risk of oversimplifying the person’s identity, for example, or the need for templates to be flexible and inclusive of the person’s evolving narrative. It could also incorporate perspectives on the relational aspects of life story work, recognising the role of family members and care staff in co-creating and sustaining the narratives of those with dementia.

Ultimately, the proposed guide would standardise life story work in dementia care across Sweden, ensuring every individual with dementia receives care that is person-centred and informed by an understanding of their life history and identity. Potentially, this could improve the quality of care while supporting the dignity and well-being of individuals with dementia, thus aligning with the broader goals of Swedish eldercare policy.

Guidelines for the design of life story templates should cover the following points:

1. Be clear about whose story it is.
The perspective of a family member or of the person with dementia him/herself?
2. Be clear about who will use the information and how.
Spell out the function of the life story so it is clear both for the person filling it in and for the person reading the information.
3. Identify other documents in use.
A review of other documents in use in the organisation helps establish the landscape in which the life story is supposed to operate.
4. Focus on the person’s life history rather than their preferences.
Information about personal preferences in food and activities are subject to change as a result of dementia or because of personal growth and development. Preferences are usually listed in other documents and should not be merged with the life story.

The lack of direction is of particular interest because life stories shape the perception of people with dementia and construct realities in dementia care homes. This study highlights the need to develop ethical guidelines for what information to include in life story guidelines intended for use in care homes.

## Limitations

This study has one significant limitation: our findings speak to the specificities of the Swedish context, and particularly the fact that only individuals with extensive care needs are eligible for placement in residential care facilities. This limitation has several implications for the generalisability and applicability of the study’s conclusions. The high threshold for admission to residential care suggests that the templates studied concern a subset of Swedish care users with the most severe impairments. This selection bias impacts the relevance of the findings to other contexts where residential care may be more readily available to those with less extensive care needs.

The Swedish approach to eldercare, which emphasises a strong welfare system and considerable public involvement, may not be directly translatable to countries with different care models. For example, in countries where private care is the norm or where there is greater reliance on family caregivers, the use and design of life story templates might differ.

The cultural norms and values that influence Swedish care practices are deeply embedded in the study’s context. Norms and values shape the expectations and interactions between care recipients, their families, and care providers. Future research would benefit from a comparative approach, examining how life story templates are used in a variety of care settings across different countries, thus offering a more comprehensive understanding of their utility and adaptability in diverse care contexts.
